# Disinfection Performance of a Drinking Water Bottle System With a UV Subtype C LED Cap Against Waterborne Pathogens and Heterotrophic Contaminants

**DOI:** 10.3389/fmicb.2021.719578

**Published:** 2021-09-03

**Authors:** Richard M. Mariita, Sébastien A. Blumenstein, Christian M. Beckert, Thomas Gombas, Rajul V. Randive

**Affiliations:** ^1^Crystal IS Inc., an Asahi Kasei Company, Green Island, NY, United States; ^2^Purgaty gmbh, Vienna, Austria

**Keywords:** cholera, disinfection, *Escherichia coli*, *Pseudomonas aeruginosa*, public health, UVC LED, *Vibrio cholerae*, water treatment

## Abstract

The purgaty One systems (cap+bottle) are portable stainless-steel water bottles with UV subtype C (UVC) disinfection capability. This study examines the bottle design, verifies disinfection performance against *Escherichia coli*, *Pseudomonas aeruginosa*, *Vibrio cholerae*, and heterotrophic contaminants, and addresses the public health relevance of heterotrophic bacteria. Bottles were inoculated with deliberately contaminated potable water and disinfection efficacy examined using colony forming unit (CFU) assay for each bacterial strain. The heterotrophic plate count (HPC) method was used to determine the disinfection performance against environmental contaminants at day 0 and after 3days of water in stationary condition without prior UVC exposure. All UVC irradiation experiments were performed under stationary conditions to confirm that the preset application cycle of 55s offers the desired disinfection performance under-tested conditions. To determine effectiveness of purgaty One systems (cap+bottle) in disinfection, inactivation efficacy or log reduction value (LRV) was determined using bacteria concentration between UVC ON condition and controls (UVC OFF). The study utilized the 16S ribosomal RNA (rRNA) gene for characterization of isolates by identifying HPC bacteria to confirm if they belong to groups that are of public health concern. Purgaty One systems fitted with Klaran UVC LEDs achieved 99.99% inactivation (LRV4) efficacy against *E. coli* and 99.9% inactivation (LRV3) against *P. aeruginosa*, *V. cholerae*, and heterotrophic contaminants. Based on the 16S rRNA gene analyses, the study determined that the identified HPC isolates from UVC irradiated water are of rare public health concern. The bottles satisfactorily inactivated the target pathogenic bacteria and HPC contaminants even after 3days of water in stationary condition.

## Introduction

The low quality of potable water is a major issue in travel medicine, especially when visiting places with poor hygienic conditions due to waterborne diseases, which pose substantial health risk (Ericsson et al., 2002). Waterborne pathogens, predominantly of fecal origin, can be transmitted *via* contaminated drinking water (Ashbolt, 2004). Even in developed countries, they represent a risk to recreational travelers who have to rely on surface water (Ericsson et al., 2002). For instance, in the United States, it is estimated that each year 560,000 people suffer from severe waterborne diseases due to the consumption of contaminated drinking water, with 7.1million suffering from mild to moderate infections, resulting in estimated 12,000 deaths a year (Medema et al., 2003). Hikers and campers are also exposed to waterborne disease risks if they consume untreated water from rivers and lakes (Schlosser et al., 2001). Diarrheal infections are a major inconvenience in the wilderness during hiking or camping and can easily spread *via* contaminated water supplies and from person-to-person.

One way to prevent waterborne diseases for healthy travelling in regions with unsafe or underdeveloped water sources is by ensuring adequate supply of potable water. Alternatively, outdoor enthusiasts can use portable and germicidal devices that ensure inactivation of microbial contaminants. UV irradiation in the UVC range (200–280nm) has demonstrated effective inactivation of microbial contaminants in water (Umar et al., 2019). Specifically, as part of the effort to accelerate the sustainable development goals (SDGs) such as clean water and sanitation goal (SDG #6; Fagunwa and Olanbiwoninu, 2020), regulating microbial load is required to control waterborne diseases caused by microorganisms, such as *Pseudomonas aeruginosa*, *Escherichia coli*, and *Vibrio* spp. (Cabral, 2010).

Cholera, caused by *Vibrio cholerae* remains a serious risk in emerging economies where sanitation is poor, health care limited, and drinking water unsafe (Heidelberg et al., 2000). Additionally, due to global warming, there is association between the spread of pathogenic vibrios and emergence of human diseases toward the temperate world (Vezzulli et al., 2016). Existing water infrastructure including electronic faucets can act as reservoirs and sources of outbreaks once contaminated. Hospital water, for instance, can disseminate opportunistic pathogens such as *P. aeruginosa* and fecal coliforms, where *E. coli* is a key species (Kanamori et al., 2016).

Furthermore, heterotrophic bacteria are a concern in drinking water systems if the counts are consistently >500 colony forming units per milliliter (CFU/ml). They can be an indication of general decrease in water quality and potential biofilm formation in municipal water (Lizzadro et al., 2019). Therefore, to eradicate elevated levels of heterotrophic plate count (HPC) and other pathogens, portable bottle devices with disinfection features can act as a form of disinfectant.

With more than 1billion people globally having no access to potable water, and 2.4billion people still living in areas without adequate sanitation systems (World Health Organization, 2000), there is need for portable, durable, appealing, personal, and highly germicidal devices to help curb enteric pathogens. Use of UVC radiation is one of the disinfection methods recognized by WHO (World Health Organization, 2000). Unlike most methods, UV disinfects by striking the target microorganism with sufficient dose of energy, while neither altering the water, nor providing any residue (World Health Organization, 2000). UV is subdivided into three distinct bands: UVA with a wavelength of 315–400nm, UVB with 280–315nm, and UVC with 100–280nm (World Health Organization, 2014). The UVC region has been found to be effective against waterborne microorganisms such as *E. coli* ATCC 25922 and *Staphylococcus aureus* ATCC 25923 (Timmermann et al., 2015), where UV systems such as Mountop Water Purifier Bottle and SteriPEN Water Purifier Kit have been used for the disinfection of *Mycobacterium abscessus*, *M. avium*, and *M. chimaera* (Norton et al., 2020). Specifically, the wavelength range between 250 and 270nm is strongly absorbed by the nucleic acids (DNA and RNA) of microbial cells (Dai et al., 2012). The use of UVC for point-of-use (POU) is technically possible at 270nm as demonstrated in a study that utilized *E. coli* K12 ATCC W3110 and *Enterococcus faecalis* ATCC 19433 (Lui et al., 2016). This study further revealed that the 310 and >455nm LEDs offer no significant UVC disinfection efficacy. Theoretically, the disinfection performance of a UVC device is a function of the intensity of UVC light (irradiance) and time of exposure resulting in a UVC dose. Greater disinfection efficacy is expected at higher UVC dose (Gora et al., 2019).

The purpose of this study was to investigate the disinfection performance of the recent commercial development of the portable purgaty One system (cap+bottle) by analyzing test bottles against pathogens and heterotrophic contaminants. This study was carried out using US municipal drinking water supplied by Cohoes Water Department in New York State.

## Materials and Methods

**Figure 1:**
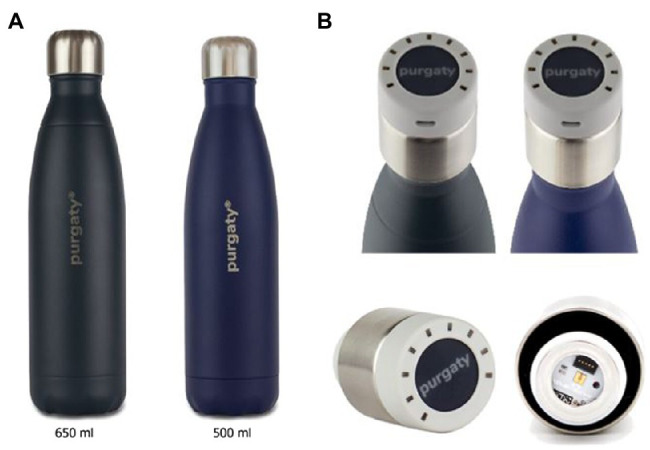
**(A)** Test bottles whose ordinary caps can be replaced with UV subtype C (UVC) emitting purgaty brain **(B)** The UVC emitting purgaty brain threaded onto both bottles (top) and shown individually from a side view and from the bottom (bottom).

There are two bottle types of purgaty One on the market, a 650 and a 500ml version. The purgaty brain (cap) can fit either of the bottles. The cap is rechargeable (Figure 1B).

### Disinfection Cap (Purgaty Brain) Design

**Figure 2:**
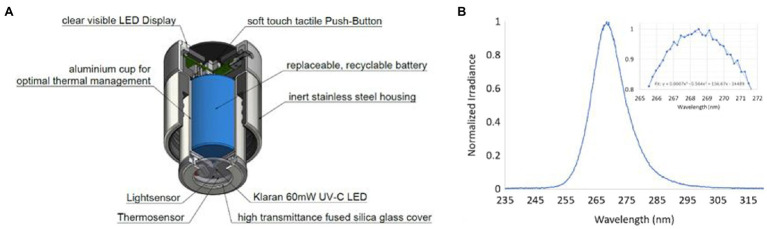
**(A)** The purgaty brain (cap emitting UVC) schematic and **(B)** measured optical spectrum of the UVC LED emission from the cap. Inset: zoom into the peak wavelength range. A trend line has been added to the data to determine the peak wavelength value.

The stainless-steel bottles (Figure 1A) are fitted with a 60mW UVC LED purgaty brain (Figure 1B) with an emission angle of 130° that offered continuous disinfection for 55s and stainless steel that exhibited reflectivity of 25%. The purgaty brain is operated by means of a button on the top of the housing (Figures 1B, 2A). Pressing the button once puts the device in standby mode, while the current charge level of the integrated battery is displayed. A second press of the button for 2s activates the preset disinfection cycle for duration of 55s. The unit is equipped with a safety feature, which allows the disinfection cycle to be started only after the cap has been correctly placed on the bottle (Figure 2A). A light sensor on the Printed Circuit Board (PCB) next to the UVC LED detects ambient light and interrupts the activated UVC LED in case of unthreading the cap or system damage with light entrance during a running disinfection cycle. This mechanism is designed to protect the user from contact with UVC radiation on skin or eyes. During the cycle, the LED and the sensor are periodically monitored to prevent malfunction. At the end of the 55s cycle, the purgaty brain flashes to indicate the end of the treatment process. The cap has one Klaran UVC LED (part number KL265-50U-SM-WD) that emits radiation at 268.5nm peak wavelength (Figure 2B) as confirmed using an Ocean Optics USB4000 photospectrometer.

The thermal design is crucial for lifetime management (irreversible power degradation) as well as for ensuring the effective optical output power level (reversible thermal derating) of the UVC LED for achieving the target disinfection performance. In the purgaty brain, an aluminum cup is used to transfer the heat from the PCB to the outer stainless-steel shell, which releases the heat to the ambient air. This design ensures that after a continuous disinfection cycle of 55s, the temperature on the aluminum PCB on which the UVC LED is mounted, does not exceed 45°C at an ambient temperature of 25°C maintaining enough optical output power levels for reliable disinfection performances. A plan-parallel 2mm thick fused silica window covers the UVC LED and seals the electronics section from water and humidity. The LED package is located at 2mm distance from the window.

### Bacterial Cultivation and Enumeration of Microorganisms

Three strains of *E. coli* The American Type Culture Collection (ATCC) 8739, *P. aeruginosa* ATCC 15442, and *V. cholerae* ATCC 25872 were obtained from ATCC (Manassas, VA, United States). Stock cultures for *E. coli* and *V. cholerae* were propagated in ATCC Medium 3: nutrient agar or nutrient broth. *Pseudomonas aeruginosa* was propagated in ATCC Medium 18: Trypticase Soy Agar/Broth. All strains were plated and incubated at 37°C for 24h. One isolated colony was picked using sterile inoculation loop and used to inoculate 25ml broth. Flasks with side baffles were used to enhance aeration. Cultures were incubated for 18–20h at 37°C while shaking at 180rpm. Culture storage was done at −80°C (0.7ml of culture with 0.7ml of sterile 40% Glycerol stock). To obtain working cultures, the microorganisms were obtained from −80°C, streaked onto corresponding agar, and incubated under same conditions. Storage of test cultures was done at −4°C.

For UVC disinfection experimental use, each strain was harvested by centrifugation at 4,000rpm for 10min. The pellets were washed using 1× phosphate buffered solution (PBS) three times for 10min. Between each wash, the supernatant was discarded, and the remaining pellet re-suspended by vortexing. After washing thrice, the pellet was resuspended in 1× PBS and used to spike dechlorinated test water to achieve a final UV transmittance (UVT) value of 96% (contaminated drinking water). Dechlorination of test water was verified with a Hach DPD Free Chlorine colorimetric test. All the other test water characteristics were within the NSF/ANSI 55 standard (Supplementary Tables S1–S3). The standard covers UVC disinfection systems within the range of 240 and 300nm for POU and point-of-entry (POE) applications (NSF International Standard, 2019).

### Disinfection Experiments

The inactivation efficiency of the purgaty One system was evaluated by inoculating the bottles with 650 or 500ml of contaminated water with a UVT of 96%. A single preset disinfection cycle of 55s was applied, following the manufacturer’s instructions on how to use the tactile push-button of the purgaty brain. Positive control bottles had no UVC activated as there was no use of the push-button, whereas negative control bottles contained uninoculated potable test water. The samples were thoroughly mixed after disinfection, serially diluted, and processed for plating.

### Decontamination of Heterotrophic Plate Count Bacteria

The HPC is an analytic method used to measure the variety of bacteria mostly found in water. HPC bacteria have no health effects at lower concentrations, instead at lower concentrations in drinking water, they are an indication of a water system well maintained. Experiments on inactivation of heterotrophic contaminants using purgaty One system were conducted on day 0 (water was not left in stationary condition), and after 3days of water being in stationary condition in bottles without prior UVC exposure. The standard HPC technique was used and incubation at 22°C and 37°C was applied using R2A agar (Gensberger et al., 2015).

### HPC Isolation, Identification, and Phylogenetic Analyses

Characteristic colonies (Table 1) were picked from the R2A agar plates and re-streaked for purity, incubated at 22°C prior to being shipped for sequencing. Submitted colony samples underwent a crude Sodium hydroxide lysis and were directly used in PCR amplification. PCR amplification was performed according to Genewiz proprietary protocol. Following amplification, enzymatic cleanup was performed prior to primer extension sequencing (GENEWIZ, Inc., South Plainfield, NJ, United States) using the Applied Biosystems BigDye version 3.1. The reactions were then run on an Applied Biosystem’s 3730xl DNA Analyzer. The primer set used in this study amplifies regions V1–V9 of the 16S gene, which is roughly a 1,400 base pairs amplicon. Internal sequencing primers were utilized in order to allow for the generation of a consensus sequence with the forward and reverse traces. Consensus files were quality trimmed to remove the N’s. The generated 16S ribosomal RNA (rRNA) gene sequences were then compared with those obtained from the NCBI database, using the program BLASTN 2.2.27+.^1^ For phylogenetic analysis, multiple alignment of *Acinetobacter* 16S rRNA gene sequences using ClustalW algorithm and tree was constructed using MEGA-X (Kumar et al., 2018). The sequences from type strains used for phylogenetics were retrieved from GenBank (National Centre for Biotechnology Information),^2^ except for the 16S rRNA gene sequences obtained through this study. *Psychrobacter cryohalolentis* K5 (Accession no. NR_075055.1) was used for rooting.

**Table 1:** Identification of the heterotrophic plate count (HPC) bacterial isolated from this study using 16S ribosomal RNA (rRNA) gene.

Strain name	Colony characteristics	Accession	Organism	Closet relative	% Similarity	Phylum
Poff1	White	MW167646	*Acinetobacter johnsonii*	*A. johnsonii* ATCC 17909	99.18	Proteobacteria
Poff2	Yellowish	MW167647	*Methylibium petroleiphilum*	*M. petroleiphilum* PM1	99.86	Proteobacteria
Poff3	White	MW167648	*Acinetobacter johnsonii*	*A. johnsonii* ATCC 17909	99.39	Proteobacteria
Pon1	Pink	MW167649	*Methylorubrum populi*	*M. populi* BJ001	99.65	Proteobacteria
Pon2	Yellowish	MW167650	*Sphingomonas ursincola*	*S. ursincola* DSM 9006	99.72	Proteobacteria
Pon3	White	MW167651	*Brevundimonas nasdae*	*B. nasdae* W1-2B	99.28	Proteobacteria
Pon4	White	MW167652	*Bradyrhizobium yuanmingense*	*B. yuanmingense* NBRC 100594	99.86	Proteobacteria
Pon5	Pink	MW167653	*Brevibacillus choshinensis*	*B. choshinensis* NBRC 15518	99.45	Firmicutes
Pon6	White	MW167654	*Brevibacillus nitrificans*	*B. nitrificans* DA2	99.39	Firmicutes
Pon7	Pink	MW167655	*Roseomonas mucosa*	*R. mucosa* MDA5527	99.72	Proteobacteria
Pon8	White	MW167656	*Brevibacillus nitrificans*	*B. nitrificans* DA2	99.25	Firmicutes
Pon9	Pink	MW167657	*Methylorubrum rhodesianum*	*M. rhodesianum* NCIMB 13779	99.23	Proteobacteria
Pon10	White	MW167658	*Cupriavidus lacunae*	*C. lacunae* S23	98.84	Proteobacteria
Pon11	White	MW167659	*Caulobacter segnis*	*C. segnis* ATCC 21756	99.43	Proteobacteria

Poff, purgaty study isolates obtained from UVC off conditions (No UVC applied). These were the most abundant. Pon, purgaty isolates obtained from UVC on conditions (UVC applied).

## Results

### Disinfection Performances Against *Escherichia coli* ATCC 8739, *Pseudomonas aeruginosa* ATCC 15442, and *Vibrio cholerae* ATCC 25872

Table 2 shows the purgaty One system effectiveness against test strains for both bottle volume types. In all cases, both test bottles obtained a log reduction value (LRV) greater than 3 (equivalent to greater 99.9% reduction) against target microbes. Additionally, the study revealed that *E. coli* ATCC 29425 was more susceptible to UVC at 268.5nm wavelength compared to other test strains (Table 2; Supplementary Tables S1 and S2). In general, the 500ml bottle obtained slightly better disinfection performances compared to the 650ml bottle.

**Table 2:** Purgaty One bottles disinfection performances against bacterial strains and environmental contaminants.

Test strain	500ml bottle’ LRV	650ml bottle’ LRV
*Escherichia coli* ATCC 29425	4.36	4.17
*Pseudomonas aeruginosa* ATCC 15442	3.56	3.24
*Vibrio cholerae* ATCC 25872	3.34	3.31
HPC day zero at 22°C	4.44	4.40
HPC day zero at 37°C	3.96	3.36
HPC after 3days of stationary at 22°C incubation	3.95	3.53
HPC after 3days of stationary at 37°C incubation	3.62	3.11

### Disinfection Performance Against HPC Bacteria

Heterotrophic plate count bacteria were present in all untreated water samples with concentrations ranging from 5.0×10^5^ to 5.67×10^5^CFU/ml (Supplementary Table S3). Disinfection using the purgaty One system reduced the microbial load of heterotrophic bacteria by >LRV3 (99.9% reduction, Table 2; Supplementary Table S3). Additionally, there was HPC bacterial selection by UVC exposure. The isolation of bacterial strains from UVC irradiated water is not new, as some strains obtained in this study have been isolated previously (Oguma et al., 2018).

### Identification and Phylogenetic Analysis of HPC Bacteria

Eleven bacterial monocultures with distinct characteristics isolated from UVC on condition were selected for molecular identification. They were identified as: *Methylorubrum populi*, *Sphingomonas ursincola*, *Brevundimonas nasdae*, *Bradyrhizobium yuanmingense*, *Brevibacillus choshinensis*, *Brevibacillus nitrificans*, *Roseomonas mucosa*, *Methylorubrum rhodesianum*, *Cupriavidus lacunae*, and *Caulobacter segnis* (Table 1). Two most abundant isolates were however obtained under non-UVC treated conditions (UVC not applied). These were identified as *Acinetobacter johnsonii* and *Methylibium petroleiphilum*. These two strains were UVC sensitive, thus accounting for the high HPC bacteria decontamination (Table 2; Supplementary Table S3). All isolates belonged to either Phylum Proteobacteria or Firmicutes (Table 1).

Taxonomic classification of HPC isolates using 16S rRNA gene identified *A. johnsonii* and *M. petroleiphilum* to be most dominant in untreated water. These two representatives of Phylum Proteobacteria were sensitive to UVC irradiation and thus not selected by UVC (+UVC condition). Strain Poff1, identified as *A. johnsonii*, which was isolated under UVC off condition (−UVC) belongs to the same Genus as *Acinetobacter baumannii*, a multidrug resistant nosocomial pathogen of global concern (Antunes et al., 2014). The study sought to confirm if the two species did not cluster together to rule out any concerns regarding the presence of *A. johnsonii* in drinking water. Phylogenetic analysis revealed that they do not cluster together (Supplementary Figure S1). Further, based on literature from previous studies, *A. johnsonii* has been confirmed to rarely cause human infections and has been found to be sensitive to virtually all antibiotics (Montaña et al., 2016).

## Discussion

Data from this study revealed >LRV3 in test bacteria, further supporting accumulating evidence for high disinfection activities of portable UVC devices (Timmermann et al., 2015). The study demonstrated high disinfection performance against *E. coli* ATCC 29425 (99.99% reduction). This can be attributed to relatively low %GC content of *E. coli* (50.68%; Engelbrecht et al., 2017) and its peak UVC sensitivity (Green et al., 2018). Although the *Vibrio* Genus has low %GC content (~47%), the presence of more elaborate DNA repair/protection processes make them less susceptible against UVC irradiation (Krin et al., 2018), thus obtaining 99.9% disinfection under similar test conditions (Table 2). This is in comparison with *P. aeruginosa* ATCC 15442, which has higher %GC content 66.17%.^3^

Even after 3days of water in stationary condition, the use of purgaty One system effectively decontaminated that HPC bacteria, obtaining <500CFU/ml, as recommended (Kohn et al., 2003). All the UVC selected bacteria are of rare to no public health concern. For instance, *B. nitrificans* is a heterotrophic nitrifying bacterium (Takebe et al., 2012) whose genera, Brevibacillus is one of the most widespread and is found in diverse environmental habitats, including drinking water (Panda et al., 2014). The study revealed that phylum Proteobacteria as most frequent of the identified isolates (Table 2). Although drinking water has complex microbiota, previous characterization studies have confirmed that phylum Proteobacteria is the most frequent in drinking water (Vaz-Moreira et al., 2017).

Results from this study indicate that UVC exposure of static water in devices such as the purgaty One systems (cap+bottle) can reduce elevated initial bacterial loads. These devices could be useful in environments where people are vulnerable to pathogens. Applications include but are not limited to those related to travel medicine, healthcare facilities where patients are vulnerable to opportunistic pathogens, hiking, remote military installations, and regions having water potability challenges.

It must be acknowledged that the current study utilized potable water that was deliberately contaminated with bacteria, and that this water, at 96% UVT, will exhibit lower absorption compared to water of low quality from natural sources like ponds which have abundant phytoplankton population (McKnight et al., 1994), iron rich lakes (Baffico, 2013), and clear alpine lakes, which ordinarily have zooplankton in the upper water layers (Tartarotti et al., 2017), and nutrient rich river estuaries or natural water with high concentrations of organic compounds (Lei et al., 2019). These waters may have UVT which is as low as 50% at 265nm within 1cm. In general, due to the different physical-chemical compositions, these natural waters will shield microorganisms from UVC, leading to lower disinfection efficacies. Additionally, in some cases, the presence of chromophoric dissolved organic matter (CDOM), for instance in arctic lakes, have spectral light attenuation and UVC absorption properties (Markager and Vincent, 2000), which may impact UVC performance. Thus, the results reported in the current study might be different if different water is used by travelers.

## Conclusion

This study investigated the efficacy of the purgaty One system (bottle+cap) on bacterial inactivation in a stationary water disinfection setup. The two types of stainless-steel water bottles (650 and 500ml) achieved more than 99.99% inactivation efficacy against *E. coli* ATCC 29425 after a single treatment cycle of 55s preset by the purgaty brain (cap), including a Klaran UVC LED with peak wavelength of 268.5nm. For *P. aeruginosa* ATCC 15442 and *V. cholerae* ATCC 25872, an inactivation efficacy of more than 99.9% was achieved. The bottles were also able to inactivate heterotrophic contaminants with more than 99.9% reduction, even after 3days of water in stationary condition in the bottles without prior exposure to UVC. These results demonstrate the ability of consistent disinfection performances of a mobile, simple-to-use and safe consumer water bottle appliance with a UVC disinfection feature including a single UVC LED only, which has extended application potential during emergency preparedness, such as for flooding situations, outdoor activities like mountain climbing, military use especially during operations in remote areas as well as consumer home use when in doubt of water potability. Lastly, these results offer fundamental evidence on how travel medicine can benefit from the use of personal UVC devices to ensure eradication of enteric pathogens.

## Data Availability Statement

The 16S rRNA gene sequences obtained from this study are available through GenBank (https://www.ncbi.nlm.nih.gov/genbank/) under the accession numbers MW167646–MW167659. The Spectral data is available *via* https://doi.org/10.6084/m9.figshare.13144538.v1.

## Author Contributions

RM was a major contributor in study design, coordinated data collection, analysis, and interpretation, and was a substantial contributor in writing the manuscript. SB contributed to study design, data analysis, and interpretation, and was a substantial contributor in writing the manuscript. CB and TG contributed to the understanding of Purgaty One System and ensured provision of study materials. RR contributed to data collection and analysis. All authors contributed to the article and approved the submitted version.

## Conflict of Interest

RM, SB, and RR work for Crystal IS Inc., an Asahi Kasei Company that manufactures UVC-LEDs. CB and TG work for purgaty, the innovators of stainless-steel drinking water bottle with cap, which inactivates microorganisms. Purgaty employees did not have any role in the microbial disinfection study design, data collection and analysis, and writing of manuscript, but contributed in the understanding of purgaty One system.

## Publisher’s Note

All claims expressed in this article are solely those of the authors and do not necessarily represent those of their affiliated organizations, or those of the publisher, the editors and the reviewers. Any product that may be evaluated in this article, or claim that may be made by its manufacturer, is not guaranteed or endorsed by the publisher.

## Footnotes

^1^ https://blast.ncbi.nlm.nih.gov/Blast.cgi

^2^ http://www.ncbi.nlm.nih.gov/

^3^ https://genomes.atcc.org/genomes/371a35eda24d4ddd
